# Potential Role of Exosomes in Cancer Metastasis

**DOI:** 10.1155/2019/4649705

**Published:** 2019-07-02

**Authors:** Wenjuan Tian, Shanshan Liu, Burong Li

**Affiliations:** ^1^Department of Clinical Laboratory, Second Affiliated Hospital, Xi'an Jiaotong University, Xi'an, Shaanxi, 710004, China; ^2^School of Medicine, Xi'an Jiaotong University, Xi'an, Shaanxi, 710061, China

## Abstract

High cancer mortality is attributed to metastasis to a large extent. However, cancer metastasis remains devoid of dynamic monitoring and early prevention in terms of current advances in diagnostic means and therapeutic modalities. Meanwhile, studies have shown that reciprocal crosstalk among cells via exosomes plays a critical role in maintaining normal physiological state or triggering disease progression, including cancer metastasis. Therefore, in this review, we focus on the latest literature (primarily from 2018) to summarize action mechanisms and experimental studies of exosomes in cancer metastasis and put forward some problems as well as new outlooks of these studies.

## 1. Introduction

Cancer is responsible for approximately 1 out of every 6 deaths and is the second-leading cause of death (following cardiovascular diseases) worldwide [1]. Meanwhile, metastases as well as their treatment consequences are the leading causes for cancer death [2]. Cancer statistics in 2019 from the American Cancer Society show the following estimates: the largest number of cancer deaths will be attributed to lung, prostate, and colorectal cancer in men. In women, lung, breast, and colorectal cancer will be largest. Moreover, the mortality of lung cancer will account for 25% of cancer deaths in 2019 [3].

Despite advances in cancer therapy, including chemoradiotherapy, immunotherapy, and molecular targeted treatment, there has yet to be satisfactory clinical outcome for patients within cancer metastasis [2, 4]. In addition, most new therapeutic strategies were developed according to their anticancer activity against tumorigenesis and primary growth, rather than their antimetastatic activity. Preclinical evidence and further clinical therapy applications of agents with antimetastatic activity are still lacking [4]. Therefore, it will be very important to develop specifically antimetastatic drug for clinical application. This will require researchers to focus their efforts on the mechanisms of cancer metastasis.

Cancer metastasis refers to the process of primary tumor cells arriving to other sites of the body, proliferating there and finally forming new tumors. It includes four main stages: intravasation (from primary tumor sites to blood vessels), extravasation (from blood circulation to future metastasis sites), tumor latency, and formation of micrometastasis and macrometastasis. The process of metastasis is modulated by epithelial-mesenchymal transition (EMT) and the reverse (MET), extracellular matrix (ECM) remodeling, activity of immune system, characteristics alteration of tumor cells, reprogramming of microenvironment cells (fibroblasts, macrophages, endothelial cells, etc.), and recruitment of bone marrow-derived cells (BMDC), such as mesenchymal stem cells (MSC) [5, 6]. In addition, the organ specificity of metastasis has gradually been unveiled by the “seed” and “soil” theory of Paget and studies of Isaiah Fidler [5]. Another intriguing finding is that organs targeted for metastasis can be altered to become suitable for tumor colonization before the arrival of cancer cells, that is, by formation of a premetastatic niche [6, 7].

Further studies have shown that exosomes play a vital role in cancer metastasis, namely, contributing in forming the premetastatic niche, influencing tumor cells and microenvironment, and determining specific organotropic metastasis [2, 4, 7]. Exosomes are formed by the inward budding of early endosomes to produce multivesicular endosomes and their fusion with cell plasma membranes [8]. They belong to the so-called extracellular vesicles (EVs) which generally include three types: apoptotic bodies, cellular microparticles / microvesicles / ectosomes, and exosomes [9]. Comparisons among the three types are shown in Table S1 of Supplementary Material [8–15]. Exosomes can transfer nucleic acids, proteins, and lipids from parent cells to recipient cells in three ways including surface receptor binding, membrane fusion with target cells, or vesicle internalization, then influencing the cell functional state [8].

Therefore, in this review, we will discuss the study of the influence of exosomes in cancer metastasis, which may provide new horizon for monitoring cancer progression, finding new therapeutic targets and realizing early intervention on metastasis.

## 2. Exosomes in Cancer Metastasis

Exosomes, serving as a cell complement, function mainly via monitoring the specific organotropism of primary tumor cells, and altering the microenvironment of targeted organs and primary tumor organs. They influence the function of tumor cells, and they change the efficacy of chemotherapy, thereby possibly functioning as dynamic monitoring biomarkers and therapeutic targets for cancer metastasis.

### 2.1. Role of Exosomes in Organ-Specific Targeting

**Figure 1:**
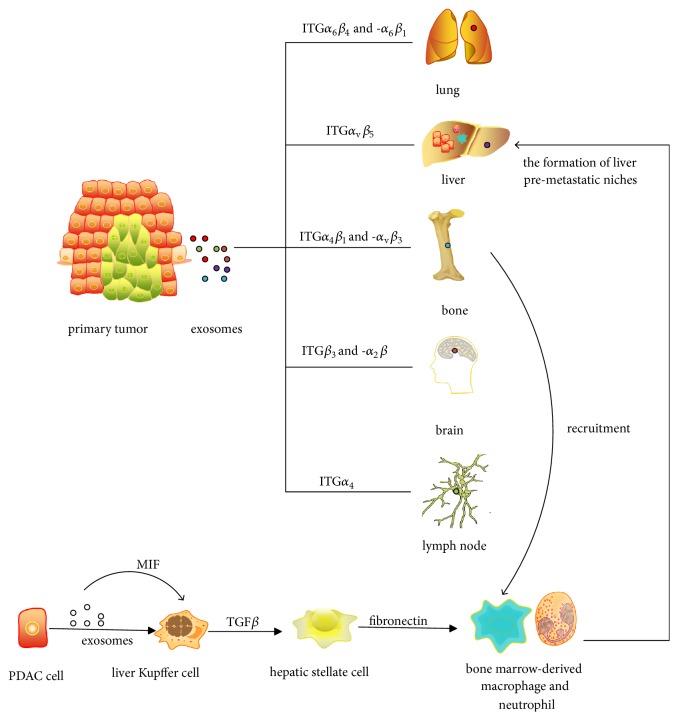
Role of exosomes in organ-specific targeting. Pancreatic ductal adenocarcinoma, PDAC.

The pioneering study from group of Prof. Layden [16] has demonstrated that exosomal integrins (ITGs) play an important role in organ-specific metastasis and colonization of tumor cells in distant sites. Their main ideas include the following. (i) tumor-derived exosomal ITGs determine the metastatic sites of the primary tumor cells; namely, exosomal ITG*α*_6_*β*_4_ and -*α*_6_*β*_1_ are associated with lung metastasis, while ITG*α*_v_*β*_5_ is associated with liver metastasis, and ITG*β*_3_ is associated with brain metastasis. (ii) These ITGs mediate the interaction of exosomes and specific resident cells of the targeted organ, namely, lung-tropic tumor-derived exosomes and lung fibroblasts and epithelial cells, liver-tropic tumor-derived exosomes and liver Kupffer cells, brain-tropic tumor-derived exosomes, and brain endothelial cells. (iii) The above interactions depend on exosomal ITGs selectively adhering to the ECM associated with specific resident cells, including laminin of lung microenvironments and fibronectin of liver microenvironments, respectively. (vi) Exosomal ITGs regulate the function of targeted cells by activating proto-oncogene tyrosine-protein kinase Src (Src) and increasing the expression of S100 (a family of genes whose symbols use the S100 prefix) gene to promote migration and inflammation. (v) Exosomal ITG content is positively associated with cancer progression. Another report from the above group has shown that pancreatic ductal adenocarcinomas (PDAC) cells-derived exosomes play a part in determining liver-tropic metastasis. These exosomes transfer migration inhibitory factor (MIF) to Kupffer cells. Thus Kupffer cells secret more transforming growth factor beta (TGF-*β*) and promote the production of fibronectin by hepatic stellate cells. Subsequently, the accumulation of fibronectin is advantageous in recruiting bone marrow-derived macrophages and forming the premetastatic niche [17]. Moreover, exosomal ITG*α*_2_*β* is also correlated with brain-tropic metastasis, while exosomal ITG*α*_4_*β*_1_ and -*α*_v_*β*_3_ promote the metastasis to bone, and exosomal ITG*α*_4_ is related to lymph node (LN) metastasis [18].  Figure 1 summarizes the above content.

### 2.2. Influence of Exosomes in Altering the Tumor Microenvironment

Tumor cells-derived and microenvironment cells-derived exosomes modify the microenvironment of the primary tumor and make targeted organ suitable for tumor progression (Table 1).

#### 2.2.1. Tumor Cells-Derived Exosomes

The tumor cells-derived exosomes transfer some crucial miRNAs, lncRNAs, and proteins to the cancer microenvironment cells, mainly containing epithelial cells, macrophages, endothelial cells, and fibroblasts. This contributes to inflammatory cell infiltration, angiogenesis, obtainment of tumor-associated cell phenotypes, and tumor innervation.

The binding of RNA to toll-like receptor (TLR) of epithelial cells or macrophages can induce tumor microenvironment inflammatory phenotypes. Liu et al. [19] have shown that exosomal small nuclear RNAs (snRNAs) of Lewis lung carcinoma (LLC) or B16/F10 melanoma cells activate TLR3 of alveolar epithelial cells and then promote chemokine release which recruits neutrophils to the lung microenvironment. Furthermore, these exosomal RNAs promote the metastasis progression by influencing the nuclear factor kappa-light-chain-enhancer of activated B cells (NF-*Κ*B) and mitogen-activated protein kinase (MAPK) pathways. In addition, it is reported that colorectal cancer (CRC) cells-derived exosomal miR-21 activates TLR7 in cytoplasm of liver macrophages. This activation results in proinflammatory phenotype transformation of macrophages with increasing expression of interleukin (IL)-6, S100 calcium-binding protein A (S100A), and matrix metalloproteinases (MMPs). Meanwhile, by a positive feedback, the above upregulated IL-6 can stimulate the expression of miR-21 mediated by signal transducer and activator of transcription 3 (STAT3) [20, 21].

The crosstalk between cancer cells and endothelial cells facilitates angiogenesis. Epithelial ovarian cancer (EOC) cells-derived exosomes enhance proangiogenic properties of human umbilical vein endothelial cells (HUVECs) via metastasis-associated lung adenocarcinoma transcript 1 (MALAT1) trafficking which may stimulate the expression of vascular endothelial growth factor (VEGF)-A, VEGF-D, epithelial-derived neutrophil-activating protein 78 (ENA-78), placental growth factor (PlGF), IL-8, angiogenin, basic fibroblast growth factor (bFGF), and leptin in HUVECs [22]. In addition, exosomal miR-25-3p from CRC cells can be internalized by HUVECs, which gives rise to decreasing expression of Krüppel-like factor 2 (KLF2) and KLF4 with the respective functions of inhibiting angiogenesis and maintaining the integrity of endothelial barrier [23]. Pessolano et al. have studied the role of exosomal annexin A1 (ANXA1) in pancreatic cancer via the MIA PaCa-2 model and knock-out technology of clustered regularly interspaced short palindromic repeats/CRISPR-associated protein 9 (CRISPR/Cas9). They have indicated that ANXA1 can elevate exosomes production. Moreover, exosomal ANXA1 can promote migration, invasion, and EMT of pancreatic cancer cells, as well as angiogenesis by interaction with HUVECs [24]. Tumor released-exosomal miR-221-3p promotes lymphangiogenesis and LN metastasis in cervical squamous cell carcinoma (CSCC) by its transmission to human lymphatic endothelial cells (HLECs), which results in the activation of miR-221-3p-vasohibin-1- (VASH1-) extracellular signal-regulated kinase (ERK)/serine/threonine-protein kinase Akt (AKT) signal axis [25].

Exosomes communicating with fibroblasts also trigger reprogramming of recipient cells into cancer-associated phenotypes. These exosomes released from lung adenocarcinoma cells (LAC) transfer miR-142-3p to lung endothelial cells and fibroblasts, which promotes angiogenesis mediated by inhibiting TGF*β*R1 in endothelial cells and induces fibroblasts tumor-associated phenotypes but may be irrelevant to TGF*β* signaling pathway [26]. Wang et al. have demonstrated that exosomal miR-27a from gastric cancer cells are also relevant to malignant transformation of fibroblasts [27].

Exosomes can also increase the nerve distribution of the microenvironment to elevate the malignant degree of tumor cells. Head and neck squamous cell carcinomas (HNSCC) released-exosomal EphrinB1 can induce tumor innervation in the PC12 neuronal model in vitro and the murine model in vivo, and patients with increased tumor innervation are prone to suffer from cancer metastasis [28].

#### 2.2.2. Tumor Associated Microenvironment Cells-Derived Exosomes

Meanwhile, surrounding stromal cells-derived exosomes are also involved in preparing microenvironment amenable for tumor colonization.

EOC-associated macrophages transfer miR-29a-3p and miR-21-5p to CD4^+^T cells via exosomes, which synergistically inhibits the activity of STAT3 and causes the imbalance of regulatory T cells (Treg)/helper T cell 17 (Th17). This contributes to form an immune-suppressive microenvironment [29].

MSCs play dual roles-stimulative or inhibitory in tumor progression by the interaction of MSC-derived exosomes and tumor microenvironment cells, which affects angiogenesis, immune response, migration, and invasion of tumors [30, 31].

### 2.3. Involvement of Exosomes in Influencing the Functions of Tumor Cells

Tumor cells- and microenvironment cells-derived exosomes commonly act on changing the proliferation activity, migration, invasion, and further distant metastasis of tumor cells (Table 2).

#### 2.3.1. Tumor Cells-Derived Exosomes

Tumor cells-released exosomes affect activities of tumor cells via autocrine and paracrine processes.

Ras-related protein Rab-27A (RAB27A) is upregulated in melanomas compared with normal skin or nevi and is related to the advanced stage of melanomas for patients. Exosomes enriched with RAB27A can rescue the invasion phenotype of the melanoma cells after the knockdown of RAB27A, which reveals that exosomes promote melanoma metastasis by changing the ability of invasion and motility of surrounding melanoma cells [32]. Exosomal lnc-matrix metalloproteinase 2-2 (lnc-MMP2-2) mediated by TGF-*β* upregulates the expression of MMP2 in lung cancer cells by its enhancer activity, which leads to increasing migration and invasion of tumor cells via the increasing vascular permeability [33]. Hypoxic CRC cells-derived exosomes promote the migration and invasion of normoxic CRC cells via protein Wnt-4- (Wnt4-) activated *β*-catenin signaling pathway, and the function depends on the hypoxia-inducible factor 1-alpha (HIF1A) expression of hypoxic cells. Upregulated HIF1A increases Wnt4 expression in hypoxic CRC cells and their released exosomes [34]. In PDAC, exosomal miR-222 transmission to cancer cells is functional to promote proliferation, invasion, and migration through two ways: (i) decreasing cyclin-dependent kinase inhibitor 1B (p27^Kip1^) (p27) expression levels directly; (ii) activating AKT by inhibition of serine/threonine-protein phosphatase 2A 55 kDa regulatory subunit B alpha isoform (PPP2R2A), which increases p27 phosphorylation and cytoplasmic p27 expression coupled with reduced nucleus expression [35]. Breast cancer cells-derived exosomal caveolin-1 (CAV1) can facilitate migration and invasion of cells with knockout of CAV1 in vitro. CAV1 is positively associated with cancer stages, which may suggest that exosomal CAV1 transferred to recipient cells promotes cancer metastasis in vivo [36].

In addition, there is a distinct model for studying exosomes function. When most studies focus on tumor-derived exosomes, Shtam et al. pay attention to exosomes from plasma of healthy donor. They have found that these exosomes can increase adhesive ability of breast cancer cells in vitro and migratory activities in Zebrafish model, which is dependent on the interaction of exosomal surface proteins and breast cancer cells, and the activation of focal adhesion kinase (FAK) signaling pathway [37].

#### 2.3.2. Tumor Associated Microenvironment Cells-Derived Exosomes

When tumor cells-derived exosomes modify diverse tumor associated microenvironment cells, in turn, these cells release exosomes acting on the functions of tumor cells.

For CRC metastasis, exosomes derived from tumor associated M2 macrophage transfer miR-21-5p and miR-155-5p to CRC cells, which results in downregulated expression of transcription activator BRG1 (BRG1) and enhanced migration and invasion of cancer cells [38]. In oral squamous cell carcinoma (OSCC), cancer-associated fibroblasts- (CAFs-) secreted exosomes deliver miR-34a-5p to cancer cells. Then miR-34a-5p activates AKT/glycogen synthase kinase-3 beta (GSK-3*β*)/*β*-catenin signaling pathway via the inhibition of tyrosine-protein kinase receptor AXL (AXL), which causes increased nuclear location of *β*-catenin and further upregulated expression of zinc finger transcription factor SNAIL (SNAIL) as well as MMP-2 and MMP-9. This finally plays an essential role in accelerating proliferation, EMT, and metastasis of cancer cells [39]. By the application of diethylnitrosamine- (DEN-) inducing long-term animal models of hepatocellular carcinoma (HCC), Alzahrani et al. have found that hepatic cancer stem cells- (CSCs-) derived exosomes function as protumor factors while bone marrow-mesenchymal stem cells (BM-MSCs) released-exosomes play an inhibitory role in tumor progression. These exosomal molecules influence apoptosis, angiogenesis, metastasis, and invasiveness as well as EMT of tumor cells via altering the expression of targeted molecules. These molecules include apoptosis regulator BAX (Bax), cellular tumor antigen p53 (p53), apoptosis regulator Bcl-2 (Bcl2), VEGF, phosphoinositide 3-kinase (P13K), extracellular signal-regulated kinase (ERK), MMP9, tissue inhibitor of metalloproteinases 1 (TIMP1), and TGF*β*1 [40].

### 2.4. Influence of Exosomes in Changing the Efficacy of Chemotherapy

**Figure 2:**
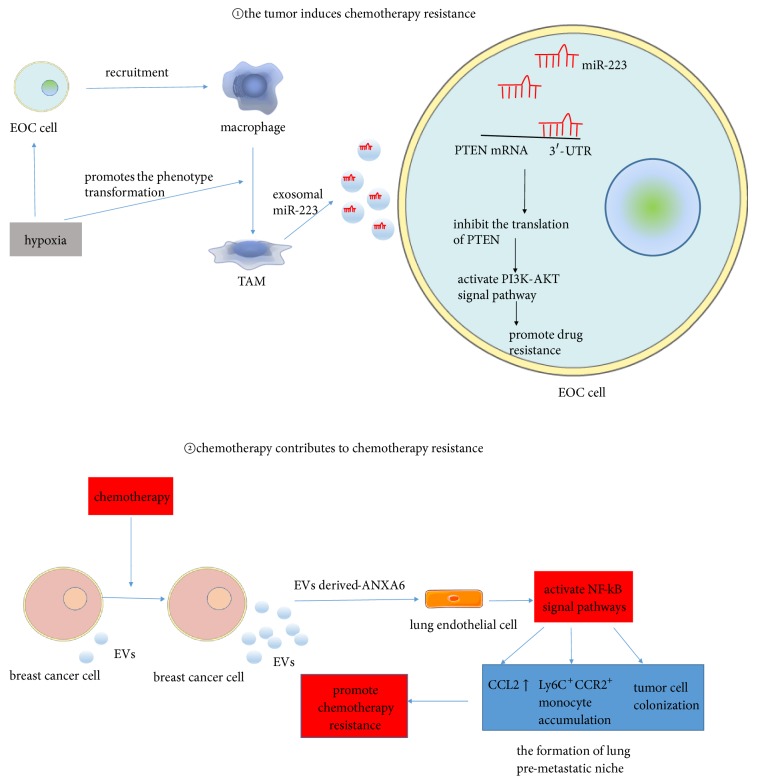
Influence of exosomes in changing the efficacy of chemotherapy. Tumor associated macrophage, TAM; epithelial ovarian cancer, EOC; ↑, upregulated.

Exosomes can transfer resistance to chemotherapy via two different ways (Figure 2): (i) the tumor induces chemotherapy resistance and, reversely, (ii) chemotherapy also promotes drug resistance.

A recent study shows that in hypoxic tumor microenvironment of EOC, tumor associated macrophages- (TAMs-) derived exosomes induce chemotherapy resistance of tumor cells via delivering miR-223 and activating miR-223/ phosphatase and tensin homolog- (PTEN-) PI3K/AKT signaling pathway [50]. In turn, chemotherapy may promote cancer metastasis. Keklikoglou et al. have demonstrated that in the breast cancer model, chemotherapy promotes the formation of lung premetastatic niche by increased release of tumor-derived EVs. These chemotherapy-stimulated EVs function as the prometastatic factor by transferring annexin A6 (ANXA6) to lung endothelial cells and then activating NF-*Κ*B signaling pathways, which causes C-C motif chemokine 2 (CCL2) upregulation, lymphocyte antigen 6C positive and C-C chemokine receptor type 2 positive (Ly6C^+^ CCR2^+^) monocyte accumulation, and tumor cells colonization in lung [51].

### 2.5. Exosomes as Potential Biomarkers of Cancer Metastasis

Some studies focus on difference analysis based on different molecular components to select exosomal biomarkers, which sets the stage for in-depth mechanism investigation (Table 3).

#### 2.5.1. Exosomal RNAs

Exosomal miR-140-3p, miR-30d-5p, miR-29b-3p, miR-130b-3p, miR-330-5p, and miR-296-3p are associated with the migration ability of hepatocarcinoma cells by the comparison analysis of exosomal miRNAs profile in fast- and slow-migrating groups of patient-derived liver cells (PDLCs). The migration ability is assessed by the wound closure percentage of wound healing assay [41]. Serum exosomal miRNA-21 and lncRNA activated by tumor growth factor-beta (lncRNA-ATB) levels in HCC patients are positively related to tumor progression [42]. miR-9 and miR-155 levels are higher in metastatic breast cancer-derived exosomes and the two miRNAs downregulate the expression of PTEN and dual specificity protein phosphatase 14 (DUSP14) in recipient cells [43]. In castration-resistant prostate cancer (CRPC), the high level of plasma exosomal miR-1290 and miR-375 is connected with poor prognosis of patients [44]. Moreover, the study of Cannistraci et al. has indicated that the expression of exosomal tyrosine-protein kinase Met (Met)/miR-130b axis in serum is related to the risk that patients with prostate cancer become resistant to castration therapy and suffer from metastasis [45]. In serum and urine of urothelial carcinoma of the bladder (UCB) patients, exosomal protein arginine N-methyltransferase 5 circular RNA (circPRMT5) levels are upregulated and associated with metastasis. The binding of circPRMT5 to miR-30c inhibits the function of miR-30c. Therefore circPRMT5 boosts EMT of UCB cells via increasing expression of SNAIL1 and reducing expression of E-cadherin, the downstream target of SNAIL1 [46].

#### 2.5.2. Exosomal Proteins

Wang et al. have shown that the level of CD82 antigen (CD82) in exosomes is negatively correlated with that in tissue for breast cancer patients, and the content of serum exosomal CD82 is higher in cancer group than that in the benign group and healthy control group. CD82 expression in serum exosomes is also positively correlated with cancer clinical stage. Therefore, there may be a redistribution of CD82 from tissue to serum exosomes, which reflects tumorigenesis and progression of breast cancer [47]. Ohshima K et al. have indicated that exosomal epidermal growth factor receptor pathway substrate 8 (Eps8) protein content is higher in metastatic cells-derived exosomes by the comparative proteome analysis of exosomes, which are purified from human pancreatic cancer cell lines with distinct stages [48]. For CRC patients with lung metastasis, studies have revealed that C-X-C chemokine receptor type 7 (CXCR7) and C-X-C motif chemokine ligand 12 (CXCL12) expression is significantly higher in metastatic site than in primary lesion, and CXCL12 expression is higher in nontumor lung tissue of patients with CRC than in control lung tissue with benign lesion. In addition, after injection of exosomes isolated from CRC cell line (CT26) into BALB/c female mice, CXCL12 expression is increased in lung tissue before cancer metastasis. Based on the above finding, the authors have stated that CRC cells-derived exosomes elevate CXCL12 expression levels in lung before metastasis [49].

The multidirectional communications of tumor cells and tumor associated microenvironment cells via the trafficking of exosomes facilitate the enhancement of malignant phenotypes of tumor cells, promote the formation of premetastatic niche, and finally exhibit clinically detectable metastasis.

In view of the important involvement of exosomes in cancer metastasis, more in-depth studies of exosomes are expected to shed more light on its biogenesis, release, and relevant functions. However, these exosome results may be questionable, due to the lack of standard isolation and characterization methods. Another disturbing factor is the fact that other EV types are likely interfering with the analysis of exosomes [9]. Indeed, the used methods currently based on size, protein composition, and morphology are not sufficient to completely separate one type of EVs from the others [8]. As is shown in Table S1, the overlap of size range occurs among the three main types of EVs. Moreover, the size range is slightly inconsistent in the literature possibly due to the various cell origin and different isolation methods among laboratories. Therefore, more standard and specific isolation and characterization methods are required for exosomes, in order to be suitable for clinical application. We refer the readers to a recent review including methodological classification, detection principle, and new technological methods for analyzing EVs [52].

Moreover, microvesicles as one of the EV types also gave rise to much attention in the cancer field. The prostate cancer cells-derived large oncosomes (a new class of shedded vesicles) are endocytosed by fibroblasts, which activates Myc proto-oncogene protein (MYC) of recipient cells via active AKT1, giving these fibroblasts a protumor phenotype [53]. Bertolini et al. have demonstrated that glioma stem cells-derived large oncosomes deliver homeobox genes and V-ATPase subunit to tumor cells and nontumor cells, which facilitates their malignant transformation [54, 55]. Therefore, the intricate identities and functions of the different EVs warrant further investigation.

## 3. Open Questions about the Influence of Exosomes on Metastasis

*(a) Are Exosomes Still Playing a Role during Tumor Latency or after Primary Tumor Resection?* During tumor latency, there are both quiescent single cells and micrometastasis. Duration of the dormant state differs in different cancers [5]. It has been well documented that metastasis sometimes still occurs after primary tumor resection.

A further question arises as to what stimulates these dormant cells into active states and promotes metastasis without a primary tumor. The contributor may be partially remaining exosomes derived from these seemingly stationary tumor cells in predetermined metastasis sites.

To demonstrate this hypothesis, it might be necessary to monitor exosomes alteration in blood of patients without detectable metastasis and then conduct long-term tracking of exosomal biomarkers for patients after tumor resection.

*(b) What Causes the Difference of Exosomal Biomarker Levels in Serum and Plasma?* Exosomal CD82 content in serum is different from that in plasma. Serum exosomal CD82 content in the malignant group is higher than that in the benign group and in the healthy group. However, the content difference between the above groups for plasma exosomes has no statistical significance; therefore serum exosome CD82 is proposed as the biomarker for breast cancer [47].

The study reminds us that detection of exosomal biomarkers in blood is dependent on selection of an appropriate specimen. Serum or plasma may give differing diagnostic test values. We need to further investigate the origin of these observed differences for a better prognosis monitoring.

*(c) What Are the Mechanisms Governing the Specific Exosomal Cargo Targeting between Tumor- and Recipient Cells Which Contribute to Inconsistent Expression of Exosomal Inclusions in Blood and Tissue? *The levels of miR-486-5p are downregulated in CRC tissue while upregulated in plasma of patients [56]. Therefore, we can postulate that redistribution of miR-485-5p from tissues to exosomes gives rise to partial expression difference between tissue and blood. Low levels of miR-486-5p in tumor cells might consequently influence cell function.

Under the above speculation, exosomes are putative molecular transporters modifying their levels both in tumor cells and in recipient cells. They further alter the state of the two kinds of cells, being either beneficial or obstructive for tumor progression. Deciphering this important question is only in its infancy.

## 4. Conclusion

It can be expected that more specific therapeutic targets for cancer metastasis will be developed following these studies. Some research has already demonstrated that tumor cells are inhibited by reducing the production of some exosomes, by interfering with their encapsulated content before or after its packaging, as well as by modifying exosomes as drug carriers [57, 58].

## Figures and Tables

**Table 1:** Influence of exosomes in altering the tumor microenvironment.

The role of tumor cells-derived exosomes in influencing the function of tumor microenvironment cells				
Donor cells	Recipient cells	Mechanisms of action	Effects	Ref.

		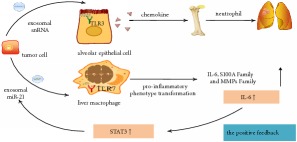	Promote ECM remodeling, the formation of inflammatory tumor microenvironment and pre-metastatic niche	
		
		
LLC or B16/F10 melanoma cells	Alveolar epithelial cells			[19]
CRC cells	Liver macrophages			[20, 21]
		
		
		

EOC cells	Umbilical vein endothelial cells (HUVECs)	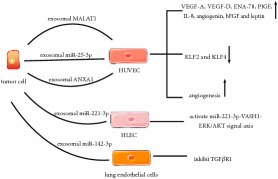	Contribute to angiogenesis	[22]
CRC cells				[23]
Pancreatic cancer cells				[24]
CSCC cells	Lymphatic endothelial cells (HLECs)			[25]
LAC cells	Lung endothelial cells			[26]

LAC cells	Fibroblasts	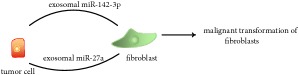	Promote the cancer-associated phenotype transformation of fibroblasts	[26]
Gastric cancer cells	Fibroblasts			[27]

		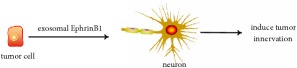		
HNSCC cells	neuronal models		Increase the nerve distribution of tumor microenvironment	[28]
			

The role of tumor microenvironment cells-derived exosomes in influencing the function of tumor microenvironment cells				

EOC-associated macrophages	CD4^+^ T cells	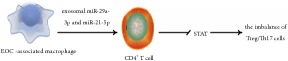	Form an immune-suppressive microenvironment	[29]

MSCs	tumor stromal cells	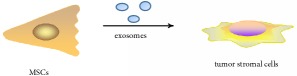	Affect angiogenesis, immune response, migration and invasion of tumor	[30, 31]

Note: Lewis lung carcinoma, LLC; Colorectal cancer, CRC; Epithelial ovarian cancer, EOC; Cervical squamous cell carcinoma, CSCC; Lung adenocarcinoma, LAC; Head and neck squamous cell carcinoma, HNSCC; Mesenchymal stem cell, MSC; Human umbilical vein endothelial cell, HUVEC; Human lymphatic endothelial cell, HLEC; ↑, Upregulated or activated; ↓, Downregulated or inhibited; 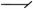, Inhibited.

**Table 2:** Involvement of exosomes in influencing the function of tumor cells.

The role of tumor cells-derived exosomes in influencing tumor cells						
Cancer type	Donor cells	Recipient cells	Study molecule	Signal axis	Effect	Ref.

Melanoma	Tumor cells	Tumor cells	RAB27A		Migration and invasion↑	[32]
Lung cancer	Tumor cells	Tumor cells	lnc-MMP2-2	lnc-MMP2-2→MMP2↑	Migration and invasion↑	[33]
CRC	Hypoxic tumor cells	Normoxic tumor cells	HIF1A	HIF1A→Wnt4-activated *β*-catenin signaling pathway↑	Migration and invasion↑	[34]
PDAC	Tumor cells	Tumor cells	miR-222	miR-222→p27↓	Proliferation, invasion and migration↑	[35]
Breast cancer	Tumor cells	Tumor cells	CAV1		Migration and invasion↑	[36]
Breast cancer	Exosomes from plasma of healthy donor(the exception of study mode)	Tumor cells	surface proteins	surface proteins→FAK signaling pathway↑	Adhesive ability and migration↑	[37]

The role of microenvironment cells-derived exosomes in influencing tumor cells						

CRC	Tumor associated M2 macrophages	Tumor cells	miR-21-5p and miR-155-5p	miR-21-5p and miR-155-5p → BRG1↓	Migration and invasion↑	[38]
OSCC	CAFs	Tumor cells	miR-34a-5p	miR-34a-5p→AXL↓→AKT/GSK-3*β*/*β*-catenin signaling pathway↑	Proliferation, EMT and metastasis↑	[39]

HCC	CSCs	Tumor cells	exosomal molecules	exosomal molecules→Bax and p53↓, Bcl2↑; VEGF↑; P13K, ERK and MMP9↑, TIMP1↓; TGF*β*1↑	Tumor progression↑	[40]
	BM-MSCs		exosomal molecules	contrary to the above expression changes	Tumor progression↓	

Note: Colorectal cancer, CRC, Pancreatic ductal adenocarcinoma, PDAC; Oral squamous cell carcinoma, OSCC; Cancer-associated fibroblast, CAF; Hepatocellular carcinoma, HCC; Cancer stem cell, CSC; Bone marrow-mesenchymal stem cell, BM-MSC; ↑, Upregulated or activated; ↓, Downregulated or inhibited.

**Table 3:** Potential exosomal biomarkers of cancer metastasis.

	Potential biomarkers	Comparison analysis	Ref.
Exosomal RNAs	miR-140-3p, miR-30d-5p, miR-29b-3p, miR-130b-3p, miR-330-5p, miR-296-3p	Exosomes derived from fast- and slow-migrating groups of PDLCs	[41]
	miRNA-21 and lncRNA-ATB	Serum exosomes isolated from patients with different HCC stages	[42]
	miR-9 and miR-155	Exosomes derived from breast cells with different metastatic ability	[43]
	miR-1290 and miR-375	Plasma exosomes derived from CRPC patients with different prognosis	[44]
	miR-130b and Met	Serum exosomes isolated from prostate cancer patients and healthy donors	[45]
	circPRMT5	Serum and urine exosomes from normal people and patients with UCB	[46]

Exosomal proteins	CD82	Exosomes derived from tissue, serum, and plasma in breast cancer patients	[47]
	Eps8	Exosomes purified from human pancreatic cancer cell lines with distinct stages	[48]
	CXCR7 and CXCL12	Exosomes isolated from tissues of primary tumor, lung metastasis, and benign lung disease in CRC patients	[49]

Note: Patient-derived liver cell, PDLC; Hepatocellular carcinoma, HCC; Castration-resistant prostate cancer, CRPC; Urothelial carcinoma of the bladder, UCB; Colorectal cancer, CRC.

## Abbreviations

### List 1 (Abbreviations of Cancer Cells, Microenvironment Cells, Cell Components, Organs, and Biological Processes)

BMDC: Bone marrow-derived cell
BM-MSC: Bone marrow-mesenchymal stem cell
CAF: Cancer-associated fibroblast
CRC: Colorectal cancer
CRPC: Castration-resistant prostate cancer
CSC: Cancer stem cell
CSCC: Cervical squamous cell carcinoma
ECM: Extracellular matrix
EMT: Epithelial-mesenchymal transition
EOC: Epithelial ovarian cancer
EV: Extracellular vesicle
HCC: Hepatocellular carcinoma
HLEC: Human lymphatic endothelial cell
HNSCC: Head and neck squamous cell carcinoma
HUVEC: Human umbilical vein endothelial cell
LAC: Lung adenocarcinoma cell
LLC: Lewis lung carcinoma
LN: Lymph node
MET: Mesenchymal-epithelial transition
MSC: Mesenchymal stem cell
OSCC: Oral squamous cell carcinoma
PDAC: Pancreatic ductal adenocarcinoma
PDLC: Patient-derived liver cell
TAM: Tumor associated macrophage
Th17: Helper T cell 17
Treg: Regulatory T cell

#### List 2 (Abbreviations of Different Molecular Components)

AKT: Serine/threonine-protein kinase Akt
ANXA1: Annexin A1
ANXA6: Annexin A6
AXL: Tyrosine-protein kinase receptor AXL
Bax: Apoptosis regulator BAX
Bcl2: Apoptosis regulator Bcl-2
bFGF: Basic fibroblast growth factor
BRG1: Transcription activator BRG1
CAV1: Caveolin-1
CCL2: C-C motif chemokine ligand 2
CCR2^+^: C-C chemokine receptor type 2 positive
CD82: CD82 antigen
CircPRMT5: Protein arginine N-methyltransferase 5 circular RNA
CRISPR/Cas9: Clustered regularly interspaced short palindromic repeats/CRISPR-associated protein 9
CXCL12: C-X-C motif chemokine ligand 12
CXCR7: C-X-C chemokine receptor type 7
DEN: Diethylnitrosamine
DUSP14: Dual specificity protein phosphatase 14
ENA-78: Epithelial-derived neutrophil-activating protein 78
Eps8: Epidermal growth factor receptor pathway substrate 8
ERK: Extracellular signal-regulated kinase
FAK: Focal adhesion kinase
GSK-3*β*: Glycogen synthase kinase-3 beta
HIF1A: Hypoxia-inducible factor 1-alpha
IL: Interleukin
ITG: Integrin
KLF: Krüppel-like factor
lnc-MMP2-2: Lnc-matrix metalloproteinase 2-2
lncRNA-ATB: LncRNA-activated by tumor growth factor-beta
Ly6C^+^: Lymphocyte antigen 6C positive
MALAT1: Metastasis-associated lung adenocarcinoma transcript 1
MAPK: Mitogen-activated protein kinase
Met: Tyrosine-protein kinase Met or Hepatocyte growth factor receptor
MIF: Migration inhibitory factor
MMP: Matrix metalloproteinase
MMP2: Matrix metalloproteinase 2
MYC: Myc proto-oncogene protein
NF-kB: Nuclear factor kappa-light-chain-enhancer of activated B cells
p27: Cyclin-dependent kinase inhibitor 1B (p27^Kip1^)
p53: Cellular tumor antigen p53
PI3K: Phosphoinositide 3-kinase
PIGF: Placental growth factor
PPP2R2A: Serine/threonine-protein phosphatase 2A 55 kDa regulatory subunit B alpha isoform
PTEN: Phosphatase and tensin homolog
RAB27A: Ras-related protein Rab-27A
S100: A family of genes whose symbols use the S100 prefix; the “S100” symbol prefix is derived from the fact that these proteins are soluble in 100% ammonium sulfate at neutral pH
S100A: S100 calcium-binding protein A
SNAIL: Zinc finger transcription factor SNAIL
snRNA: Small nuclear RNA
Src: Proto-oncogene tyrosine-protein kinase Src
STAT3: Signal transducer and activator of transcription 3
TGF*β*: Transforming growth factor beta
TIMP1: Tissue inhibitor of metalloproteinases
TLR: Toll-like receptor
VASH1: Vasohibin-1
VEGF: Vascular endothelial growth factor
Wnt4: Protein Wnt-4.

## Additional Points

*MIA PaCa-2.* The cell line was established by A. Yunis et al. in 1975 from tumor tissue of the pancreas obtained from a 65-year-old Caucasian male. The information is obtained via ATCC website (https://www.atcc.org/).* Tumor Associated M2 Macrophage. *Macrophages generally consist of the two types: M1- and M2 macrophages. Studies have shown that M2 macrophages are more likely to promote tumor progression.

## Supplementary Materials

Supplementary Materials Table S1: Difference among the three main types of EVs.
