# Supplementary material for: Potential Role of Exosomes in Cancer Metastasis
Source: Biomed Res Int. 2019 Jul 2;2019:4649705. doi: 10.1155/2019/4649705 (PMC6634128; doi:10.1155/2019/4649705)
Supplement: Supplementary Materials — Table S1: Difference among the three main types of EVs. [file 4649705.f1.pdf]

Supplementary Materials

Table S1: Difference among the three main types of EVs

	Exosomes	Microvesicles	Apoptotic bodies
Size	30-100nm (10, 11, 15)<br>50-150 (16)<br>40-100nm (13)<br>40-120nm (14)<br>40-200nm, blood exosomes (12)<br>30-200 (53)	50-500nm or up to 1μm (16)<br>up to 1000nm or relatively small vesicle with 100nm (13)<br>50-1000nm (14)<br>150-1000nm (11)<br>100-1000nm (10)	500-2000nm (14)
Origin	Endosomal system	Plasma membrane	Plasma membrane
Budding process	Inward	Outward	Outward
Other names	Prostasomes (13)<br>Tolerosomes/Dexosomes /Nanovesicles/Exosome-like vesicles (16)	Platelet dust (16)<br>Microparticles (9, 11, 13)<br>Ectosomes (9, 11, 13)<br>Oncosomes (16)<br>Shedding vesicles (11, 13)	
Release process	Released after multivesicular endosome fusion with the plasma membrane	Shedding from plasma membrane	Released by plasma membrane blebbing during cell apoptosis
